# The morphing of geographical features by Fourier transformation

**DOI:** 10.1371/journal.pone.0191136

**Published:** 2018-01-19

**Authors:** Jingzhong Li, Pengcheng Liu, Wenhao Yu, Xiaoqiang Cheng

**Affiliations:** 1 School of Resource and Environmental Sciences, Wuhan University, Wuhan, Hubei, China; 2 College of Urban and Environmental Science, Central China Normal University, Wuhan, Hubei, China; 3 Faculty of Information Engineering, China University of Geosciences, Wuhan, Hubei, China; 4 Faculty of Resources and Environmental Science, Hubei University, Wuhan, Hubei, China; China University of Mining and Technology, CHINA

## Abstract

This paper presents a morphing model of vector geographical data based on Fourier transformation. This model involves three main steps. They are conversion from vector data to Fourier series, generation of intermediate function by combination of the two Fourier series concerning a large scale and a small scale, and reverse conversion from combination function to vector data. By mirror processing, the model can also be used for morphing of linear features. Experimental results show that this method is sensitive to scale variations and it can be used for vector map features’ continuous scale transformation. The efficiency of this model is linearly related to the point number of shape boundary and the interceptive value *n* of Fourier expansion. The effect of morphing by Fourier transformation is plausible and the efficiency of the algorithm is acceptable.

**Table:** 

Symbol Legend		
Symbol	Name	Definition
*s*_1_	Scale	A large scale
*s*_2_	Scale	A small scale
*s*_*g*_	Scale	A medium scale between *s*_1_ and *s*_2_
*α*	Vector graph	The vector graph at scale *s*_1_
*β*	Vector graph	The vector graph at scale *s*_2_
*r*_*g*_	Vector graph	The vector graph at scale *s*_*g*_
*f*_1_	Function	Shape signature function of *α*
*f*_2_	Function	Shape signature function of *β*
*f*	Function	Shape signature function of *r*_*g*_
*g*	Parameter	A normalized weight parameter about *s*_*g*_
*S*	Length	Path length of curve
*S*_*i*_	Length	The *i*^*th*^ point’s path length along curve
*X*_*i*_	X coordinate	The *i*^*th*^ point’s x coordinate
*Y*_*i*_	Y coordinate	The *i*^*th*^ point’s y coordinate
*X*(*s*)	X coordinate	The x coordinate at length s
*Y*(*s*)	Y coordinate	The y coordinate at length s
*N*	Number	Point number of curve
*K*	Parameter	An integer
*n*	Parameter	The interceptive value
*A*_*X*,*n*_	Parameter	Fourier transformation parameter
*B*_*X*,*n*_	Parameter	Fourier transformation parameter
*A*_*Y*,*n*_	Parameter	Fourier transformation parameter
*B*_*Y*,*n*_	Parameter	Fourier transformation parameter
*OD*	Parameter	Overlap degree of two shapes
*area*	Function	Area function
*P*_*i*_	Polygon	The *i*^*th*^ polygon
*B*_1_	Vector graph	The vector building footprint at large scale
*B*_2_	Vector graph	The vector building footprint at small scale
*H*_1_	Vector graph	The vector lake boundary at large scale
*H*_2_	Vector graph	The vector lake boundary at small scale
*L*_1_	Vector graph	The vector open curve at large scale
*L*_2_	Vector graph	The vector open curve at small scale

## 1 Introduction

Morphing is an important technique in computer graphics and computer vision [1]. In general, morphing can be defined as a gradual and smooth transformation of one key shape into another [2]. Traditional vector morphing involves two basic processes: characteristic correspondence and shape interpolation [3]. The purpose of characteristic correspondence is to establish a characteristic matching relationship between the initial and the target graphs, and the purpose of shape interpolation is to implement a smooth gradient from the initial graphic to the target graphic on the basis of the matching relationship.

In the field of computer graphics, there are two kinds of methods to determine the characteristic correspondence of vector graphics. One is to establish global characteristic correspondence based on optimization technology, and the other is to establish local correspondence based on shape similarity measurement [4]–[6]. As the geometry and topology characteristics of the graphs need to be consistent in the interpolation process, a lot of work has been done on shape interpolation. Sederberg et al [6] proposed an intrinsic interpolation method, which can avoid the shrinkages by both interpolating of the edge length and the point angle of two input shapes. As-rigid-as-possible interpolation method was presented to improve the effect of boundary interpolation by rigid motion and compatible triangulations [7, 8].

In the field of cartography and Geographical Information System (GIS), morphing technology can be used to realize continuous generalization and multi-scale representation of spatial data [9]. For the characteristic matching problem, Cecconi and Galanda [10] built a one-to-one correspondence between two graphics by inserting additional points based on the proportional chord-length principle. Nöellenburg et al. [11] presented a dynamic programming based matching method to get the global feature correspondence. Deng and Peng [12] split the two linear features into pairs of subpolylines and got the correspondence relationship between the subpolylines by shape similarity measurement. A simulated annealing based optimization algorithm for characteristic matching and shape interpolation was discussed in [13]. To match the orthogonal boundary characteristics, Li et al. [14] proposed a turning function based matching method for building footprints’ continuous generalization. For the trajectories problem of shape interpolation, straight-line trajectories are the most popular method. Whited and Rossignac [15] used ball-maps to detect corresponding points for two smooth objects, and then five different trajectories–hat, linear, tangent, circular and parabolic–were used for morphing.

Whether in the computer graphics or GIS fields, it is difficult to establish the characteristic correspondence relationship, especially when the boundaries of two anchor shapes are totally different. To avoid the problem of characteristics matching, an alternative strategy is to transform two input geometric graphics into two implicit functions and generates the intermediate shapes by fusing the implicit functions with different parameters. For example, Hughes [16] presented a scheduled Fourier transform to perform morphing in which both basic states were represented as a combined function of high frequency and low frequency, and the morphing effect was achieved by the fusion of the two functions. He et al. [17] decomposed the two basic states into a set of frequency wavelet bands, applied a smooth interpolation to each band, and reconstructed the results to generate the intermediate state. This paper proposes a morphing method of geographical features based on Fourier transformation, which does not require the process of characteristic correspondence. The basic idea of morphing by Fourier transformation is to transform the vector coordinates of geographical feature in spatial domain into function expression in frequency domain using Fourier transformation, then a weighted combination of Fourier expressions of two vector shapes is used to construct the functional expression of the intermediate shape, finally an inverse transformation is performed on the intermediate functional to get the intermediate shape.

The rest of paper is organized as follows. Section 2 investigates the methodology of morphing model by Fourier transformation. The experiments on different geographical features are given in Section 3. Section 4 presents conclusions with suggestions for future improvements.

## 2 Methodology of morphing model by Fourier transformation

### 2.1 Conceptual model

In mathematics a two-dimensional vector boundary can be converted into a one-dimensional real function or complex function based on a certain shape signature [18]–[20]. The vector expressions of area and linear geographic features in GIS can also be converted into a one-dimensional function. Assuming that *α* and *β* are two vector representations of the same geographical entity at large scale *s*_1_ and small scale *s*_2_, their corresponding function expressions are *f*_1_ and *f*_2_ respectively, and then the function of intermediate representation *r*_*g*_ can be represented as:

$$\begin{array}{c} f=\left(1-g\right)\times f_{1}+g\times f_{2}, \end{array}$$ (1)

In Formula (1), *f* is a composite function based on *f*_1_ and *f*_2_, and *g* is a normalized weight parameter related to intermediate scale. Assuming that the interpolation scale is *s*_*g*_, then the relationship between *g* and scales can be expressed as the following formula.

$$\begin{array}{c} g=\frac{s_{1}-s_{g}}{s_{1}-s_{2}} \end{array}$$ (2)

Because of *s*_2_ ≤ *s*_*g*_ ≤ *s*_1_, so 0 ≤ *g* ≤ 1. The greater the value of *g*, which means the smaller the scale *s*_*g*_, then the function *f* is more similar to *f*_2_; the smaller the value of *g*, which means the greater the scale *s*_*g*_, then the function *f* is more similar to *f*_1_. But how to establish the reciprocal transformation relationships between *α* and *f*_1_, *β* and *f*_2_, and *r*_*g*_ and *f*. Fourier transformation has the property of reversibility besides the invariance to rotation, translation, and scaling [19]–[23]. Here we use Fourier transformation to implement conversions between geometry coordinates and shape signature function.

### 2.2 Fourier transformation of vector curve: A brief review

Fourier transformation is a way to represent a periodic function or periodic signal into the sum of a set of simple oscillating functions, namely sines and cosines. In GIS, discrete Fourier transformation and inverse transformation usually use an equal-distance sampling approach to represent cartographic curve [24]–[27]. Actually simple area object (polygon without inner ring) can be considered as the “trajectory” formed by a point object’s periodic motion. The coordinates of “trajectory” can be described as(X(s), Y(s)), where X(s) and Y(s) are the segmental functions of path length *S* which can be expressed as Eqs (3) and (4).

$$\begin{array}{c} X\left(s\right)=X_{i}+\frac{X_{\left(i+1\right)}-X_{i}}{S_{\left(i+1\right)}-S_{i}}\times \left(S-S_{i}\right) \end{array}$$ (3)

$$\begin{array}{c} Y\left(s\right)=Y_{i}+\frac{Y_{\left(i+1\right)}-Y_{i}}{S_{\left(i+1\right)}-S_{i}}\times \left(S-S_{i}\right) \end{array}$$ (4)

Where (*X*_*i*_, *Y*_*i*_) is the coordinate of the *i*^*th*^ point on the curve, *S*_*i*_ is the path length from the start point to the *i*^*th*^ point along the curve, *S* is the moving point’s path length from the start point, and *S*_*i*_ ≤ *S* ≤ *S*_(*i*+1)_ and 0 ≤ *i* ≤ *N* − 1. Given the total point number of a closed curve is *N*, the function X(s) and Y(s) are periodical functions with a period of *S*_*N*_. Then the functions can be expressed as Eqs (5) and (6) by Fourier series [20]:

$$\begin{array}{c} X\left(s\right)=\lim_{K\to \infty }[\sum_{n=0}^{K}\left(A_{X,n}\cos2n\pi s+B_{X,n}\sin2n\pi s\right] \end{array}$$ (5)

$$\begin{array}{c} Y\left(s\right)=\lim_{K\to \infty }[\sum_{n=0}^{K}\left(A_{Y,n}\cos2n\pi s+B_{Y,n}\sin2n\pi s\right] \end{array}$$ (6)

Where *K* is an integer, *n* is the interceptive value of describing the Fourier expansion of curve needed, and

$$\begin{array}{c} A_{X,n}=\sum_{i=0}^{N-1}\int_{S_{i}}^{S_{i+1}}\left[X_{i}+\frac{X_{i+1}-X_{i}}{S_{i+1}-S_{i}}\left(S-S_{i}\right)\right]\cos2n\pi sds \end{array}$$ (7)

$$\begin{array}{c} B_{X,n}=\sum_{i=0}^{N-1}\int_{S_{i}}^{S_{i+1}}\left[X_{i}+\frac{X_{i+1}-X_{i}}{S_{i+1}-S_{i}}\left(S-S_{i}\right)\right]\sin2n\pi sds \end{array}$$ (8)

$$\begin{array}{c} A_{Y,n}=\sum_{i=0}^{N-1}\int_{S_{i}}^{S_{i+1}}\left[Y_{i}+\frac{Y_{i+1}-Y_{i}}{S_{i+1}-S_{i}}\left(S-S_{i}\right)\right]\cos2n\pi sds \end{array}$$ (9)

$$\begin{array}{c} B_{Y,n}=\sum_{i=0}^{N-1}\int_{S_{i}}^{S_{i+1}}\left[Y_{i}+\frac{Y_{i+1}-Y_{i}}{S_{i+1}-S_{i}}\left(S-S_{i}\right)\right]\sin2n\pi sds \end{array}$$ (10)

Eqs (7)–(10) are known as the Fourier transformation which converts a two-dimensional closed curve into a one-dimensional function, and Eqs (5) and (6) are the inverse Fourier transformations which can reconstruct the curve. Generally, the larger the value of *n*, the smaller the values of *A*_*X*,*n*_, *B*_*X*, *n*_, *A*_*Y*, *n*_ and *B*_*Y*, *n*_ are. When *n* approaches to infinite, *A*_*X*,*n*_, *B*_*X*,*n*_, *A*_*Y*,*n*_ and *B*_*Y*,*n*_ will approximately be zero. In other words, the larger the value of *n*, the closer the Eqs (5) and (6) are to the original curve.

### 2.3 Morphing model by Fourier transformation

According to Formulas (1), (5) and (6), shape signature functions *f*_1_, *f*_2_ and *f* can be denoted as follows:

$$\begin{array}{c} f_{1}:\{\begin{array}{cc} X_{\alpha }\left(s\right)= & \lim_{K\to \infty }[\sum_{n=0}^{K}\left(A_{X_{\alpha },n}\cos2n\pi s+B_{X_{\alpha },n}\sin2n\pi s\right]\\ Y_{\alpha }\left(s\right)= & \lim_{K\to \infty }[\sum_{n=0}^{K}\left(A_{Y_{\alpha },n}\cos2n\pi s+B_{Y_{\alpha },n}\sin2n\pi s\right] \end{array} \end{array}$$ (11)

$$\begin{array}{c} f_{2}:\{\begin{array}{cc} X_{\beta }\left(s\right)= & \lim_{K\to \infty }[\sum_{n=0}^{K}\left(A_{X_{\beta },n}\cos2n\pi s+B_{X_{\beta },n}\sin2n\pi s\right]\\ Y_{\beta }\left(s\right)= & \lim_{K\to \infty }[\sum_{n=0}^{K}\left(A_{Y_{\beta },n}\cos2n\pi s+B_{Y_{\beta },n}\sin2n\pi s\right] \end{array} \end{array}$$ (12)

$$\begin{array}{c} f:\{\begin{array}{cc} X(S) & =\left(1-g\right)X_{\alpha }\left(S\right)+gX_{\beta }\left(S\right)\\ Y(S) & =\left(1-g\right)Y_{\alpha }\left(S\right)+gY_{\beta }\left(S\right) \end{array} \end{array}$$ (13)

From the above discussion, the value of *n* determines the approximation degree of the Fourier expansion series to the original shape. If we specify the value of approximation degree in advance, the value of *n* can also be determined through a certain way. First of all, we need to define a function to measure the similarity between two shapes. Assume that the original and intermediate shapes are *r*_0_ and *r*_*g*_ (The *r*_*g*_ is at intermediate scale and generated by Fourier transformation)respectively, and their intersection is *r*_0∩*g*_. We can define the approximation degree of two shapes as follows:

$$\begin{array}{c} OD=\frac{area(r_{0\cap g})}{area(r_{0})} \end{array}$$ (14)

The parameter *OD* ranges from 0 to 1. The closer the value is to 1, the higher the accuracy of the approximation. Then *OD* can be considered as the accuracy of original shape expression by Fourier series. While given a specific *OD* value, the minimal *n*, which can firstly reach the *OD*, is chosen as the interceptive value. For the same *OD*, the shape *r*_*α*_ and *r*_*β*_ will be approximated with different *n* values because of their different shape complexity. According to the Formula (13), the morphing model has only one value of *n*. Here we choose the bigger *n* as the common interceptive value of the two source shapes *α* and *β*.

For linear features in GIS, their boundaries are often expressed as open curves. Although open curves can also be expressed as piecewise functions, they are not periodical functions. Therefore, the linear features cannot be directly fitted by Fourier series. To express the open curve with Fourier series, we can expand the piecewise function into periodic function by mirroring the open curve through the line that connects the first and the last points of the curve [20]. Based on the closed curve, the same processes of extracting Fourier coefficients and inverse Fourier transformation can be applied as described above. But the range of *S* is $\left[0,\frac{S_{n}}{2}\right]$ instead of [0, *S*_*n*_] for open curve.

## 3 Experimental analysis

We design three sets of experiments to verify the feasibility and effectiveness of the morphing model by Fourier transformation. The first set of experiments are designed to validate the feasibility of Fourier function representation of vector data. Especially we focus on the discussion of truncation parameter *n* and its’ variation with different geometry shapes. The second set of experiments are used to validate the feasibility of the morphing model based on Fourier transformation for continuous scale transformation of map data. We validate the experiment by using three kinds of data such as orthogonal boundary area features, smooth boundary area features and smooth boundary linear features. The third set of experiments are used to validate the effectiveness of the morphing model based on Fourier transformation by contrast experiments.

### 3.1 The experiment of Fourier series representation of vector data

**Fig 1:**
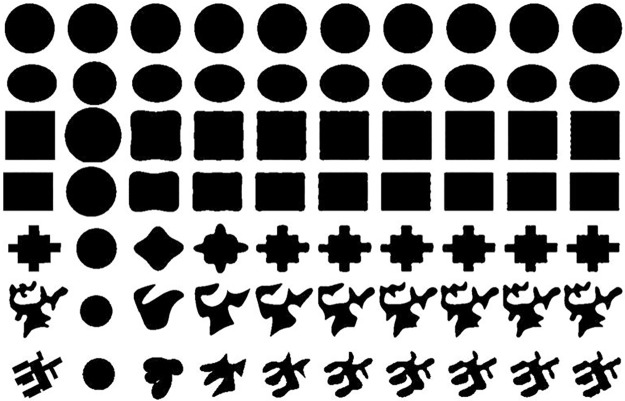
The polygon approximation by Fourier descriptors for different shape types at different interceptive values.

To illustrate the feasibility of Fourier function representation of vector data, we use seven sets of experimental data to perform the experimental verification. The experimental data includes circle, ellipse, square, rectangle, convex polygon, concave polygon with smooth boundary and concave polygon with orthogonal boundary, which can be denoted as *P*_1_, *P*_2_, *P*_3_, *P*_4_, *P*_5_, *P*_6_, *P*_7_. In Fig 1, the leftmost polygons on each column are *P*_1_ to *P*_7_, and the remaining 9 polygons on each column are approximated by Fourier series with different interceptive value *n*. From left to right, the values of *n* are 1, 5, 9, 13, 17, 21, 25, 29 and 33 respectively. Table 1 is the approximation degree between the original polygon and the fitting polygon by Fourier expansion with different interceptive value *n*. The Fourier expansion method is based on Formula (5) and (6), and the approximation degrees are calculated by Formula (14).

**Table 1:** The degrees of approximation (OD) of different shapes at designated interceptive values.

	1	5	9	13	17	21	25	29	33
OD of *P*_1_	0.999	0.999	0.999	0.999	0.999	0.999	0.999	0.999	0.999
OD of *P*_2_	0.912	0.999	0.999	0.999	0.999	0.999	0.999	0.999	0.999
OD of *P*_3_	0.907	0.978	0.990	0.994	0.996	0.997	0.998	0.998	0.999
OD of *P*_4_	0.883	0.974	0.988	0.993	0.995	0.997	0.998	0.998	0.999
OD of *P*_5_	0.852	0.894	0.952	0.983	0.987	0.989	0.990	0.992	0.995
OD of *P*_6_	0.642	0.711	0.819	0.867	0.913	0.949	0.958	0.971	0.979
OD of *P*_7_	0.616	0.696	0.804	0.852	0.891	0.903	0.926	0.953	0.969

From Table 1 we can see that the interceptive parameter *n* is sensitive to the morphological characteristics of vector data. Generally, the shapes with high compactness, high degree of convex and smooth boundary are faster to achieve the specified approximations degree *OD* with smaller *n* values. The shape of circle is the easiest one to be fitted by Fourier transformation. Meanwhile, ellipse and square can be quickly fitted by Fourier transformation with small *n* values. For example, when *OD* = 0.99, their *n* = 5 and 9. The convex polygon *P*_5_ is easier to approximate than concave polygons *P*_6_ and *P*_7_ by this method and the concave polygon with a smooth boundary *P*_6_ is easier to approximate than the concave polygon with an orthogonal boundary *P*_7_.

Generally speaking, the Fourier transformation model can capture the main outline-information of the shape in low frequency (small *n*-value) while capture the detail information in high frequency (large *n*-value). We can obtain the approximate expression of original polygon gradually through a gradual increase in the *n*-value. So this method can be used to fit the vector data in GIS.

### 3.2 The morphing of vector data by Fourier transformation

**Fig 2:**
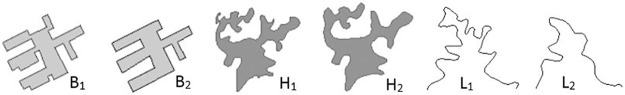
The experimental data. (Group one is building feature with orthogonal boundary. Group two is lake feature with smooth boundary. Group three is linear feature with smooth boundary).

The main application of morphing model in cartography and GIS are continuous generalization and multi-scale representation. Here we will test the feasibility of continuous generalization of geographical features using the morphing model presented in section 2. Three type of different geographical features are used to finished this experiment, which are showed in Fig 2. They are building features with orthogonal boundary, lake features with smooth boundary and linear features with smooth boundary. Each set of experimental data consists of two representations of the same geographic entity, one corresponding to a large scale representation, and the other corresponding to a small scale representation. Obviously, the representations at large scale are more detailed than these at small scale. These line features, who’s geometry are open curves, need to be reconstructed into closed curves by mirroring. Table 2 shows the relationship between the degree of approximation OD and the interceptive value *n* of the three sets of data.

**Table 2:** The degree of approximation OD with different interceptive value *n*.

	1	5	9	13	17	21	25	29	33	37	41	45	49	53	57	61
*B*_1_	0.616	0.696	0.804	0.906	0.935	0.943	0.956	0.963	0.969	0.978	0.981	0.984	0.988	0.989	0.991	0.992
*B*_2_	0.644	0.719	0.858	0.940	0.955	0.967	0.974	0.978	0.985	0.989	0.992	0.992	0.994	0.995	0.994	0.995
*H*_1_	0.642	0.711	0.819	0.867	0.913	0.949	0.958	0.971	0.979	0.984	0.987	0.988	0.991	0.993	0.993	0.995
*H*_2_	0.661	0.723	0.837	0.885	0.935	0.968	0.977	0.984	0.988	0.993	0.995	0.996	0.997	0.998	0.998	0.999
*L*_1_	0.543	0.612	0.718	0.765	0.814	0.868	0.894	0.916	0.944	0.956	0.967	0.972	0.983	0.988	0.989	0.990
*L*_2_	0.563	0.625	0.738	0.786	0.834	0.882	0.911	0.932	0.954	0.968	0.979	0.983	0.988	0.992	0.994	0.996

**Fig 3:**
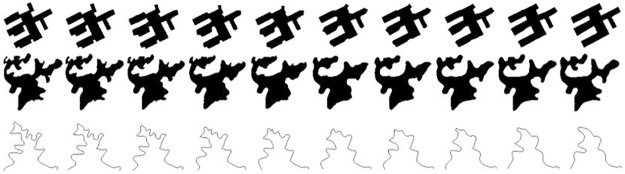
The morphing effects of different features with g interval 0.1.

**Fig 4:**
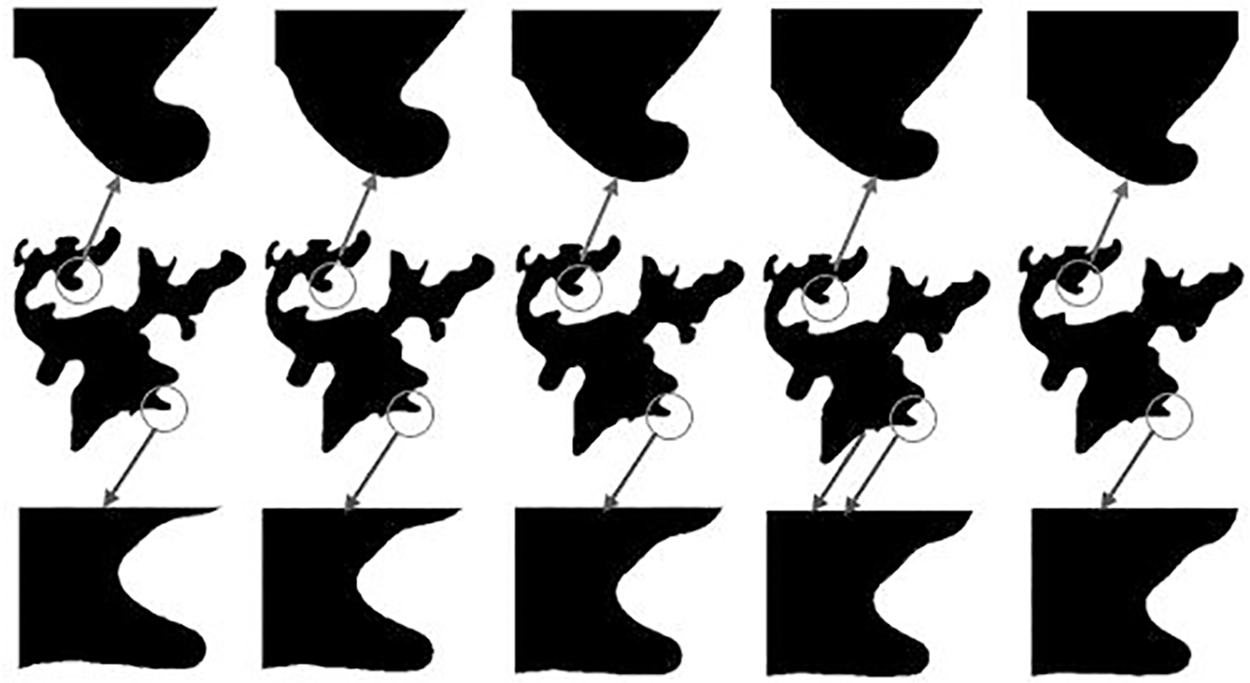
The morphing effects of lake feature with g interval 0.01.

In order to ensure the accuracy of morphing, we need to set a high *OD* value in the process of Fourier fitting of vector data, saying *OD* = 0.99. When *OD* = 0.99, we can get the related interceptive value *n* for *B*_1_, *B*_2_, *H*_1_, *H*_2_, *L*_1_, *L*_2_ are 57, 41, 49, 37, 61 and 53 respectively from Table 2. So for the morphing model in Formula (13), the parameters *n* for building feature, lake feature and linear feature are 57, 49 and 61 respectively. From the discussion in conceptual model we know that we can get representations of vector data with different resolutions by tuning the parameter *g*. To test the sensitivity of the morphing model to parameter *g*, two series of *g* are designed. One series are 0.1, 0.2, 0.3, 0.4, 0.5, 0.6, 0.7, 0.8, 0.9, 1, the interval of *g* is 0.1, and the experimental results are showed in Fig 3. The other series are 0.95, 0.94, 0.93, 0.92, 0.91, the interval of *g* is 0.01, and the results are showed in Fig 4. For convenience of observation, some details within the circle area in the second row in Fig 4 are enlarged and shown in the first and third rows.

From Fig 3 we can find that the overall trend in graphic expression of building, lake and linear features is from fine to coarse with the gradual increase of *g* value. In other words, this morphing model can reflect the gradual process of map representation from big scale to small scale, and all intermediate shapes are fused from the initial (e.g., *B*_1_, *H*_1_, *L*_1_) and final figures (e.g., *B*_2_, *H*_2_, *L*_2_). So it is a process of continuous generalization. At the same time, we also find that the model has better application effect on the feature with smooth boundary (such as lake and contour line features) than that with orthogonal boundary (such as building footprint). In the first row of Fig 3, there are some obvious non orthogonal boundary during the gradual process from *B*_1_ to *B*_2_, these results are not consistent with the specification of building footprint. For the lake and linear features with smooth boundary, their boundaries always maintain a smooth and continuous gradient during the process of interpolation, and the results are plausible.

Considering the adaptability of the morphing model to different features, the other experiment with g series of 0.95, 0.94, 0.93, 0.92, 0.91 is only conducted for the lake feature with smooth boundary. From the amplification effects in Fig 4, we can see even small changes of *g* (saying 0.01) can cause tiny but smooth, continuous gradients in geometric shapes. So this morphing model is sensitive to scale variations and it is suitable for continuous scale transformation.

### 3.3 Comparative experiment

**Fig 5:**
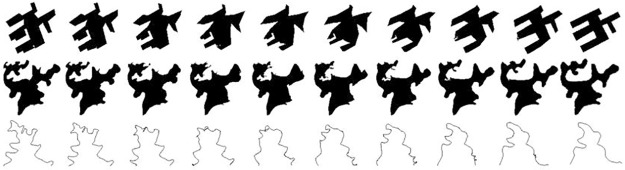
The morphing effects by linear interpolation method.

The following experiments will test the interpolation effect and time efficiency of the morphing model proposed in this paper by comparative analysis and time analysis. The experiment data is still the same as shown in Fig 2, and the *g* series is still 0.1, 0.2, 0.3, 0.4, 0.5, 0.6, 0.7, 0.8, 0.9, 1. The comparative experiment uses the linear interpolation method proposed in [10]. First we get the one-to-one correspondence between two boundary points by inserting abundant points into the boundary with less points based on the proportional chord-length principle. Then we conduct linear interpolation between the corresponding coordinate points to get the intermediate shape series. Experimental results are shown in Fig 5, from which we can see that the interpolation graph series of building, lake and contour line are abnormal and there are some cases of self-intersection at intermediate scales. Compared with the experimental effects in Fig 3, we can conclude that the morphing model proposed in this paper is better than the simple linear interpolation and the gradient effect is reasonable and effective.

For the morphing of vector data by Fourier transformation, the main time consumption includes three parts: the conversion from vector data to Fourier series by Formula (7)–(10), the generation of intermediate function by combination of the two Fourier series related the large and small scale by Formula (13), and the reverse conversion from the combination function to vector data by Formula (5) and (6). All of these processes are linear time series. So the time consumption mainly depends on two factors: the point number of shape boundary and the interceptive value *n* of Fourier expansion. The point number of boundary of *B*_1_, *B*_2_, *H*_1_, *H*_2_, *L*_1_, *L*_2_ are 37, 26, 260, 196, 152 and 93 respectively. The linear features are open curves, and the point actually participating in calculation is double of the real number because of the mirroring operation. So the point numbers of *L*_1_ and *L*_2_ should be 304 and 186.

**Fig 6:**
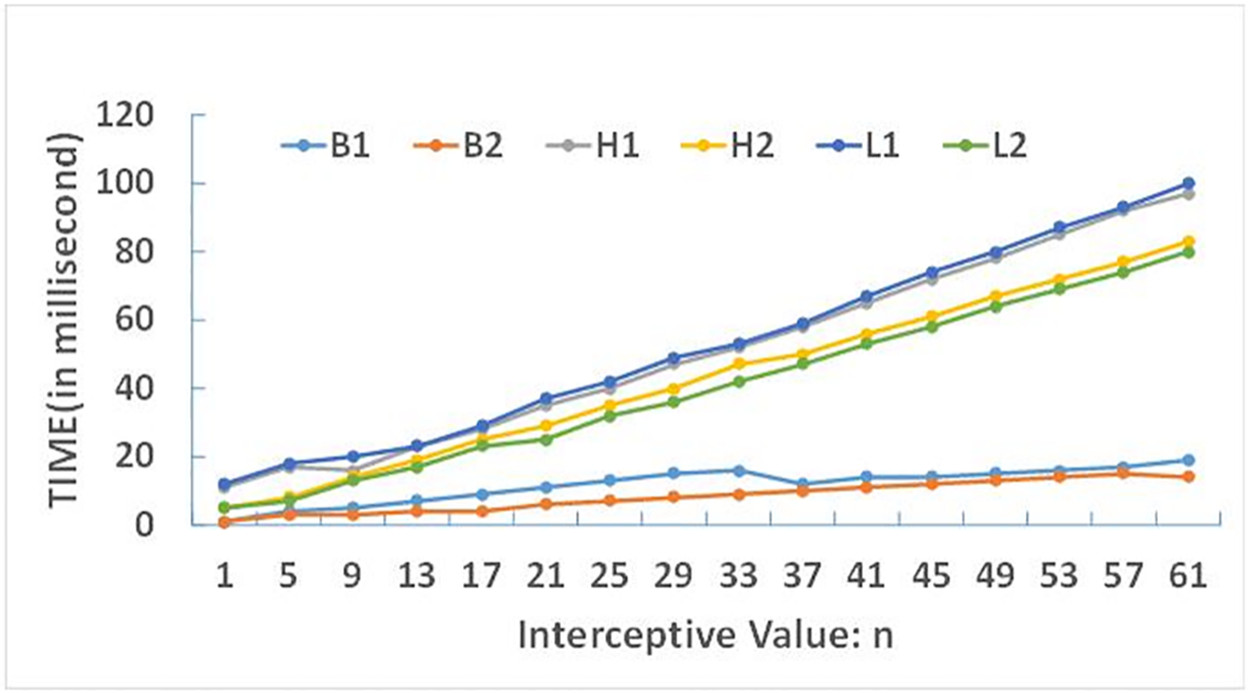
The relationship of time consumption and the interceptive value n.

**Fig 7:**
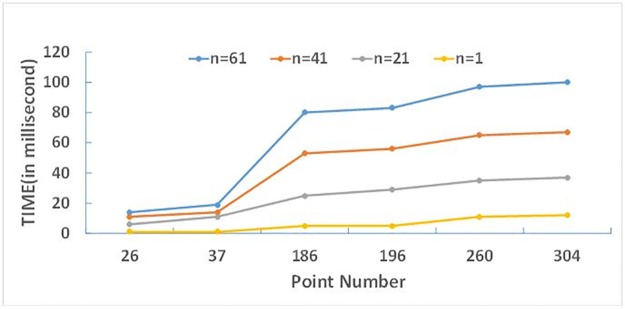
The relationship of time consumption and the number of point N.

Table 3 records the time consumption (including all three main processes) of the Fourier transformation of the six figures in Fig 2 with different interceptive value *n* from 1 to 61, the Fig 6 reflects the relationship of time consumption and the interceptive value, and the Fig 7 reflects the relationship of time consumption and the number of point. From which we can see that the time consumption is linearly related to the number of point and the interceptive value of Fourier expansion series. So the time efficiency is acceptable. For most of moderate complex map objects, the accuracy of the Fourier transformation is close to 0.99 when the interceptive value *n* = 50 is used, and time consumption for morphing by Fourier transformation is about 150 milliseconds. So the morphing model proposed in this paper can generate about seven high quality intermediate shapes of moderate complexity per second. If the shape accuracy requirement decreases, the efficiency can be further improved by using a smaller n.

**Table 3:** The time cost of morphing model by Fourier transformation.

	1	5	9	13	17	21	25	29	33	37	41	45	49	53	57	61
*B*_1_	1	4	5	7	9	11	13	15	16	12	14	14	15	16	17	19
*B*_2_	1	3	3	4	4	6	7	8	9	10	11	12	13	14	15	14
*H*_1_	11	17	16	23	28	35	40	47	52	58	65	72	78	85	92	97
*H*_2_	5	8	14	19	25	29	35	40	47	50	56	61	67	72	77	83
*L*_1_	12	18	20	23	29	37	42	49	53	59	67	74	80	87	93	100
*L*_2_	5	7	13	17	23	25	32	36	42	47	53	58	64	69	74	80

## 4 Conclusion

This paper presents a morphing model of vector geographical data based on Fourier transformation. The basic idea of the model is to transform the spatial representation of vector data into function expression in frequency domain, and then get the intermediate shapes by functions composition and inverse Fourier transformation. Subject to the constraints of periodic functions, linear objects must be closed before subsequent morphing transformation can be performed. Experimental results show that this method can be used for vector map features’ continuous scale transformation. This morphing model is sensitive to scale variations and even small changes of parameter *g* (saying 0.01) can cause tiny but smooth and continuous gradients in geometric shapes. The efficiency of this method mainly depends on the point number of shape boundary and the interceptive value *n* of Fourier expansion, and the time consumption is linearly related to these two factors. Generally speaking, there are two advantages of the proposed method compared with the previous method. First, by introducing Fourier transformation, complex characteristics points matching process can be avoided. It is a simple morphing model. Second, this model can be used for morphing transformation of both linear and area features. It is a practical morphing model.

Finally, we can conclude that the morphing model proposed in this paper is suitable for these vector map features who have smooth boundary. For those features with orthogonal boundary, their orthogonality property cannot be maintained in the morphing process. It is not suitable for the morphing of building footprint, nor can it be used for the morphing of polygon with inner island.
